# Supplementary material for: Quality Control and Authentication of Argan Oils: Application of Advanced Analytical Techniques
Source: Molecules. 2023 Feb 15;28(4):1818. doi: 10.3390/molecules28041818 (PMC9966767; doi:10.3390/molecules28041818)
Supplement: Supplementary file 1 [file molecules-28-01818-s001.zip › molecules-2152497-supplementary.pdf]

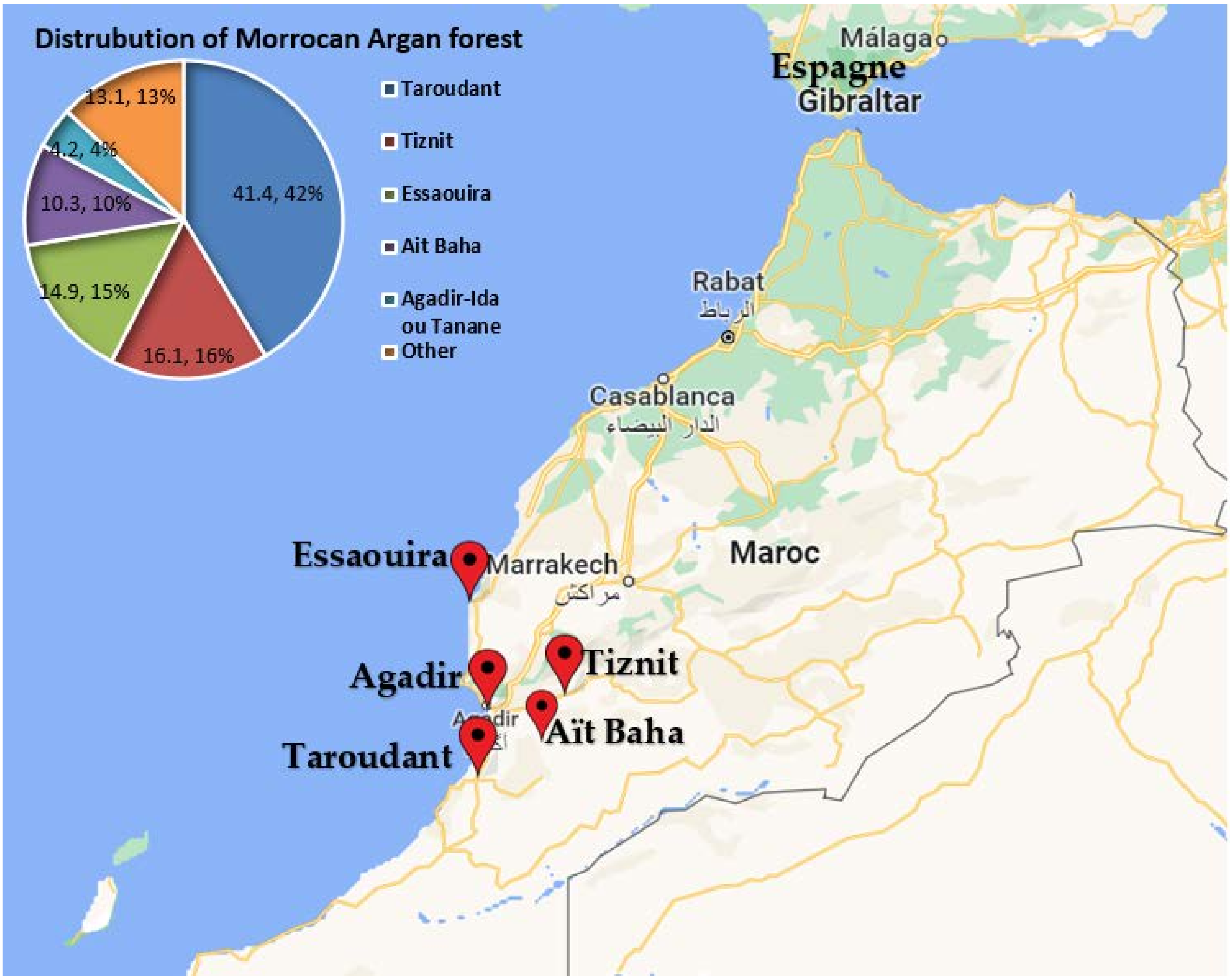


**Figure S1.** The Moroccan Argan tree regions (distribution based on [1]).

**Table S1** Percentages of total fatty acids in Argan oil according to different research studies. NA: not applicable.

| Extraction technique | Origin of oil | Fatty acids | | | | | References |
|---|---|---|---|---|---|---|---|
| | | Oleic C18:1 | Palmitic C16:0 | Linoleic C18:2 | Stearic C18:0 | Linolenic C18:3 | |
| Traditional | NA | 43.0 - 49.1 | 11.5 - 15.0 | 29.3 - 36.0 | 4.3-7.2 | ≤0.3 | [85] |
| Mechanical | Agadir | 44.9 ± 0.5 | 12.7 ± 0.4 | 30.5 ± 0.7 | 34.1 ± 0.6 | 0.13 ± 0.13 | [20] |
| | Ait-Baha | 46.8 ± 0.5 | 14.1 ± 0.4 | 33.1 ± 0.5 | 6.8 ± 0.3 | 0.29 ± 0.04 | |
| | Ait-Baha | 43.9 ± 0.5 | 14.1 ± 0.4 | 30.3 ± 0.8 | 4.6 ± 0.2 | 0.11 ± 0.03 | |
| | Taroudant | 48.6 ± 0.5 | 14.6 ± 0.3 | 35.4 ± 0.6 | 5.6 ± 0.3 | 0.26 ± 0.02 | |
| | Tiznit | 47.3 ± 0.7 | 13.8 ± 0.4 | 34.1 ± 0.6 | 6.9 ± 0.3 | 0.25 ± 0.04 | |
| Traditional | NA | 45.0 – 50.0 | 11.8 - 13.5 | 29.1 - 34.1 | 5.2 - 6.9 | 0.03 - 0.1 | [86] |
| Traditional | Tindouf | 45.1 | 2.0 | 35.9 | 6.2 | Traces | [6] |
| NA | Taroudant | 47.2 ± 1.5 | 15.4 ± 0.7 | 30.3 ± 1.5 | 5.9 ± 0.5 | NA | [87] |
| Mechanical | Ait Baha, | 48 | 13.0 | 32.0 | 5.0 | NA | [71] |
| Traditional | Tidzi, Tiout | 47 | 13.0 | 33.0 | 5.0 | NA | |
| Mechanical | Agadir | 46.8 ± 0.5 | 12.8 ± 0.3 | 33.8 ± 0.6 | 4.9 ± 0.2 | 0.2 ± 0.05 | [75] |
| EVAO regulation limit values | | 43.0 - 49.0 | 11.5 - 15.0 | 29.3 - 36.0 | 4.3 - 7.2 | ≤0.2 | [46] |

**Table S2** Metal composition of Argan Oils determined by ICP-AES. BQL: Below Quantification Limit, NA: not applicable.

| Metal content (mg/kg) | | | | | | | | Origins | Extraction methods | References |
|---|---|---|---|---|---|---|---|---|---|---|
| Ca | Co | Cr | Fe | K | Mg | Cd | Zn | | | |
| 11.70 ± 0.03 | 0.550 ± 0.006 | 0.015 ± 0.003 | NA | 0.620 ± 0.007 | 5.95 ± 0.06 | 0.100 ± 0.003 | 0.650 ± 0.003 | Agadir | Mechanical extraction | [44] |
| 11.10 ± 0.03 | 0.390 ± 0.003 | 0.011 ± 0.003 | NA | 1.110 ± 0.004 | 7.62 ± 0.03 | 0.170 ± 0.003 | 0.400 ± 0.003 | Taroudant | | |
| 8.00 ± 0.01 | 0.410 ± 0.003 | 0.024 ± 0.003 | NA | 0.420 ± 0.008 | 4.33 ± 0.06 | 0.14 ± 0.01 | 0.530 ± 0.003 | Essaouira | | |
| 25.0 - 65.5 | 0.1 – 0.5 | 1.0 – 3.0 | 3.0 ± 1.0 | 8.6 - 11.7 | 2.2 – 18.0 | <0.26 | <0.23 | Morocco | Traditional extraction | [46] |
| 10.0 ± 0.1 | NA | BQL | 0.46 ± 0.01 | 0.51 ± 0.01 | 2.43 ± 0.03 | 0.11 ± 0.01 | 0.036 ± 0.010 | Agadir | Traditional extraction | [45] |
| 9.8 ± 0.1 | NA | BQL | 0.18 ± 0.01 | 0.43 ± 0.01 | 6.32 ± 0.05 | 0.13 ± 0.01 | 0.033 ± 0.010 | Taroudant | | |
| 6.7 ± 0.1 | NA | BQL | 0.32 ± 0.01 | 0.32 ± 0.01 | 2.82 ± 0.02 | 0.12 ± 0.01 | 0.043 ± 0.010 | Essaouira | | |
| 7.4 ± 0.9 | NA | BQL | 0.67 ± 0.05 | 1.11 ± 0.01 | 2.28 ± 0.23 | BQL | BQL | Tiznit | Traditional extraction | [88] |
